# Comparison of single- and double-spaced feeders with regard to damaging behavior in pigs

**DOI:** 10.3389/fvets.2023.1073401

**Published:** 2023-02-22

**Authors:** Roberta Maria D'Alessio, Alison Hanlon, Keelin O'Driscoll

**Affiliations:** ^1^Pig Development Department, Teagasc, Animal & Grassland Research and Innovation Centre, Moorepark, Fermoy, Ireland; ^2^UCD Veterinary Sciences Centre, University College Dublin Belfield, Dublin, Ireland

**Keywords:** growing-finishing pigs, feeder, enrichment, grass, tail biting, harmful behaviors, pig production

## Abstract

This work compared the behavior and performance of 24 groups of 12 pigs kept in pens with either a DOUBLE [one feed space aligned with the front wall of the pen (WALL), and one immediately adjacent (IN)] or SINGLE (WALL only) spaced feeder, from weaning until slaughter. Pens were equipped with a rack of fresh grass and a rubber toy (weaning phase) or a wooden board (finishing phase). Every 2 weeks, interaction with the enrichment, aggressive, harmful, and play behaviors were recorded for 5 min, four times a day. In addition, the pigs were individually assessed every 2 weeks for ear, tail, and flank injuries using a 4-point scale. The duration of feeder occupancy, feed space occupancy, number of and duration of feeding bouts, and aggressive behavior at the feeder were recorded continuously from video recordings (two times while in the weaner stage and two times while in the finisher stage, one camera/pen; 1 h per occasion). Individual body weight was recorded at weaning, transfer, and slaughter, and feed delivery was recorded daily at the pen level; from these measurements, average daily gain, feed intake, and feed conversion ratio were calculated. Data were analyzed using SAS v9.4. There was no effect of treatment on damaging behaviors. Pigs in DOUBLE had worse tail lesion scores on 6 of the 9 recording days (*P* < 0.001), but values rarely exceeded 1. Total feeder occupancy tended to be longer in DOUBLE than in SINGLE (*P* = 0.06). DOUBLE selected the IN feed space more often than WALL regarding the number of feeding bouts (*P* < 0.001). During feeding, pigs in DOUBLE received fewer aggressive behaviors than SINGLE (*P* < 0.001) and experienced fewer displacements than SINGLE (*P* < 0.001). Although both experimental groups had a similar weight at slaughter (*P* > 0.05), the FCR was lower in DOUBLE than in SINGLE (*P* < 0.05). These data suggest that doubling space at the feeder to two spaces/12 pigs reduced aggression and displacement behaviors at the feeder, indicating less competition for food. However, increasing feeder space was not a management strategy that could ensure reduced tail biting on commercial pig farms.

## 1. Introduction

Despite Council Directive 2008/120/EC banning routine tail docking of piglets, the practice continues to be widespread within the European pig sector (1). Indeed in many of the large pig-producing countries, over 95% of commercial pigs' tails are docked (2, 3). Tail docking is performed to mitigate against tail biting, which is one of the major problems in intensive indoor pig production systems (3). It is a multifactorial problem related to internal risk factors (linked to the pig, e.g. genetics, gender, age, health status, and tail length) and influenced by a great variety of external factors (linked to the environment and management such as stocking density, rooting material, ambient temperature, floor type, feeding system, and feed type) (4). Tail-biting outbreaks are difficult to predict and can occur even in farms where management practices for prevention are implemented (5, 6), as well as impacting pig welfare, carcass and lung disease lesions, decreased carcass weights, and condemnations at slaughter are possible consequences derived from this injurious behavior (7–11). As a result, farmers routinely tail dock to avoid economic losses (12).

Although tail docking acts as a preventive measure against tail biting, damaging biting behavior can be redirected toward the ears, flank, and legs (13). This can result in severe skin lesions and partial amputation of the ears in the case of ear biting in weaner and fattener pigs (14, 15). Indirectly, biting behavior can result in lameness, infections due to the wounds caused by biting (4), immunosuppression (16), reduced growth, and, in some cases, death (17).

Among the factors that are involved in this abnormal biting behavior, redirected foraging behavior has been suggested to be important. Behavioral activity budgets in a semi-natural environment show that pigs spend a high proportion of their daily time foraging (e.g., rooting and grazing) and exploring their environment (e.g., nosing, orienting to stimuli, and manipulating objects) (18). Modern intensive production systems are characterized by (partly-) slatted floors and the absence of a substrate in which the pigs can root, in contrast to the environment in which these animals have evolved (19). Exploratory behavior is shown in the absence of environmental stimuli suggesting that it is a behavioral priority (20).

The inability to access high-priority resources, such as the feeder, can also increase tail-biting risk (21). Competition for food, such as restricting meals per day or access to the feeder, will increase the potential for some pigs to become frustrated. It could also increase size variation within a group, which will further influence an individual's ability to compete for access to the feeder, and potentially result in sudden forceful biting of tails (22).

Wallenbeck et al. (23) observed an increased frequency of agonistic interactions, foraging behavior away from the feeders, and shortened feeding bouts, several weeks before the start of tail-biting outbreaks. Furthermore, a disrupted pattern of feeding behavior among pigs in groups developing tail biting was observed long before the first injured tail (23). Tail-bitten pigs are also reluctant to stand at the feeder (4), which will contribute to reduced weight gain. Increasing feeder space reduces the competition for access to feed and, thus, could also facilitate recipients of tail-biting to feed undisturbed. The use of double- or multi-space feeders has, thus, potential to contribute to tail-biting prevention, even in long-tailed pigs (24).

This study investigated the potential for double-spaced feeders to reduce harmful behaviors in static groups of piglets from weaning to slaughter by observing tail, ear, and flank biting, by recording tail, ear, and flank lesions; pig behavior around the feeder; and interaction with enrichment material, relative to single-spaced feeders.

## 2. Materials and methods

### 2.1. Ethical statements

Ethical approval was obtained from the Teagasc Animal Ethics Committee (TAEC2020-259). All procedures were carried out in accordance with Irish legislation (SI. No. 543/2012) and the EU Directive 2010/63/EU for animal experiments.

The primary ethical concern for this study was the high risk of tail biting and tail-biting outbreaks due to not docking pigs' tails. In this regard, pigs' tails were inspected two times a day during the week and one time daily during the weekend by the first author of the study (RD'A), and routine checks were also performed by the farm and technical staff in the research center.

A tail-biting outbreak was defined as when two or more pigs in a pen were observed to have fresh, clearly visible blood presented on their tail, or when one pig presented blood on the tail and most of the other tails in the pen were hanging low. When an outbreak occurred, the first intervention to stop it was to add two pieces of hessian (0.20 m × 0.20 m) and one hanging rubber toy to the pen as additional enrichment, and a layer of ointment (Cheno Unction, PharVet, Ireland) applied to bleed tails to reduce the smell of blood and bleeding. If this stopped the biting, the additional enrichment was removed after 3 days (72 h). If this did not stop the biting behavior, the suspected tail biter/s was moved to a hospital pen. In cases where pigs' tails were severely injured, victims were also removed temporarily to a hospital pen for treatment and recovery. The hospital pens were located in the same room as experimental pens, had the same dimensions, and were provided with the same enrichment. Pigs removed to hospital pens were accommodated with a partner from the same group to ease reintroduction after no more than 72 h.

### 2.2. Animals and housing

The experiment was carried out in the Pig Research Facility in Teagasc, Moorepark, Fermoy, Co. Cork, in Ireland. A total of 288 pigs [Camborough (PIC) × Tempo (Topigs Norsvin)] from weaning age (~28 days) to slaughter were used in the experiment. The experiment was replicated over time, with 144 pigs included in each replicate. Pigs were reared according to conventional commercial practice in Ireland, where male piglets are not castrated. The pigs' tails were not docked.

At weaning, piglets were individually tagged and weighed. Experimental pigs were not mixed at weaning, remaining in their litter groups. Only litters where there were at least five individuals of both sexes, and where there were at least 12 healthy piglets were used. In litters where there were more than 12 pigs, pigs that had any obvious injuries were selected for removal from the experiment. Individuals were then removed selectively so that within each replicate, six blocks of two litters (*n* = 12 pigs per litter) were created that were matched for sex ratio (11 blocks, 6F + 6M; 1 block, 5F + 7M) and weight (8.6 ± 1.49 kg). Within each block, litters were then randomly assigned to one of two treatments; either a SINGLE- or DOUBLE-spaced feeder pen. Litters from the same block were kept in pens facing each other across a central corridor.

**Figure 1:**
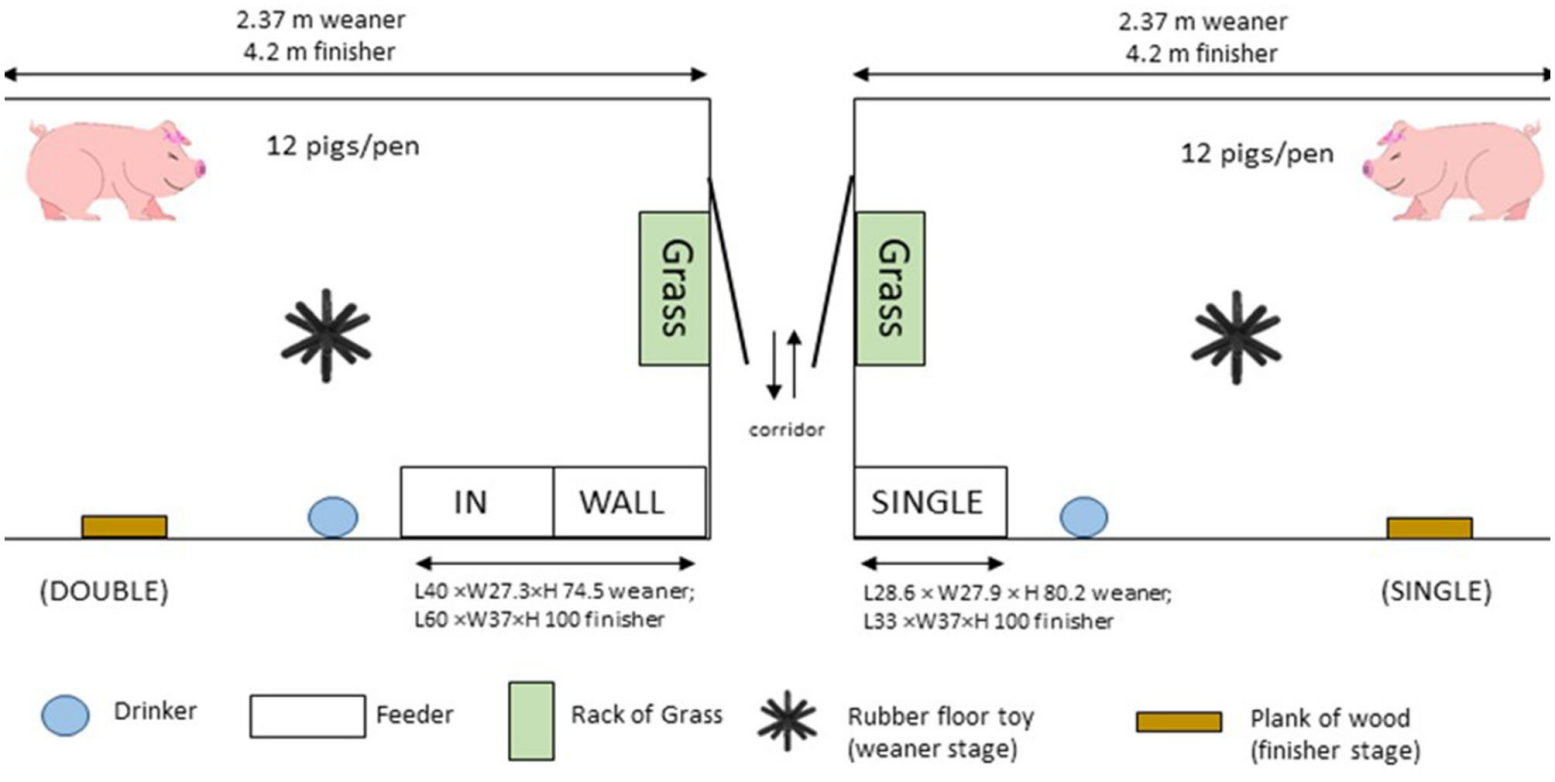
Plan view of pen and feeder layouts for both DOUBLE and SINGLE treatments. DOUBLE pens had double-spaced feeders, one called WALL, which was aligned with the front wall of the pen, and IN, which was adjacent to WALL inside the pen. SINGLE had a single-spaced feeder aligned to the front wall of the pen.

The dimensions of the weaner and fattener pens were 2.37 × 2.36 m and 4.2 × 2.4 m, respectively, including the feeder area. All pens were fully slatted, with plastic flooring in the weaner stage, and concrete in the finisher. Each pen was furnished with a nipple drinker and a wet-dry feeder which delivered *ad libitum* pelleted feed and had an integrated water nipple. SINGLE-spaced feeder pens were equipped with a feeder that permitted one pig to feed at a time. The feeder was located in the corner of the pen adjacent to the central corridor in the room and measured 28.6 × 27.9 × 80.2 cm (feeder space 25 × 25.4 × 12) in weaner pens, and 33 × 37 × 100 cm (feeder space 30.5 × 34.5 × 12.5) in finisher pens. DOUBLE-spaced feeder pens were equipped with a feeder that permitted two pigs to feed simultaneously, measuring 40 × 27.3 × 74.5 (feeder space 18.8 × 24.8 × 12) in weaner pens, and 60 × 37 × 100 (feeder space 28.8 × 34.5 × 12.5) in finisher pens. The feeder was placed in the same location in the pen as SINGLE so that one feed space was aligned with the front wall of the pen (WALL) and one was immediately adjacent (IN; Figure 1).

In the weaner housing, the temperature was kept at ~28°C immediately post-weaning and reduced by 2°C every 2 weeks thereafter, while in the fattening housing, the temperature was maintained at 20°C. To ensure a normal circadian rhythm, the light and dark cycle was kept at 12 h per day. Artificial light at ~150 lux and 130 lux in the weaner and finisher house, respectively (7:30 a.m. to 16:30 p.m.) was supplemented by natural daylight from windows. Seven weeks post-weaning (at 11 weeks of age), pigs were transferred to the finisher housing without further regrouping. Spare empty pens of the same dimension, which were present in the same room and under the same management as the experimental pens, were used as the hospital pens. When used, the hospital pens were provided with the same enrichment material used during the trial.

### 2.3. Enrichment material

The European Council requires that pigs must have permanent access to manipulable material [Art. 3 (5), Annex 1 (4)]. To comply with the legislation, two types of enrichment material were provided; either a rubber floor toy (weaner stage) or plank of wood (finisher stage) and a rack of fresh grass (Perennial Ryegrass and White Clover swards, both stages). Prior to the start of the experiment and at the end of each stage, the rubber floor toy and plank of wood were weighed to determine consumption by the pigs.

**Figure 2:**
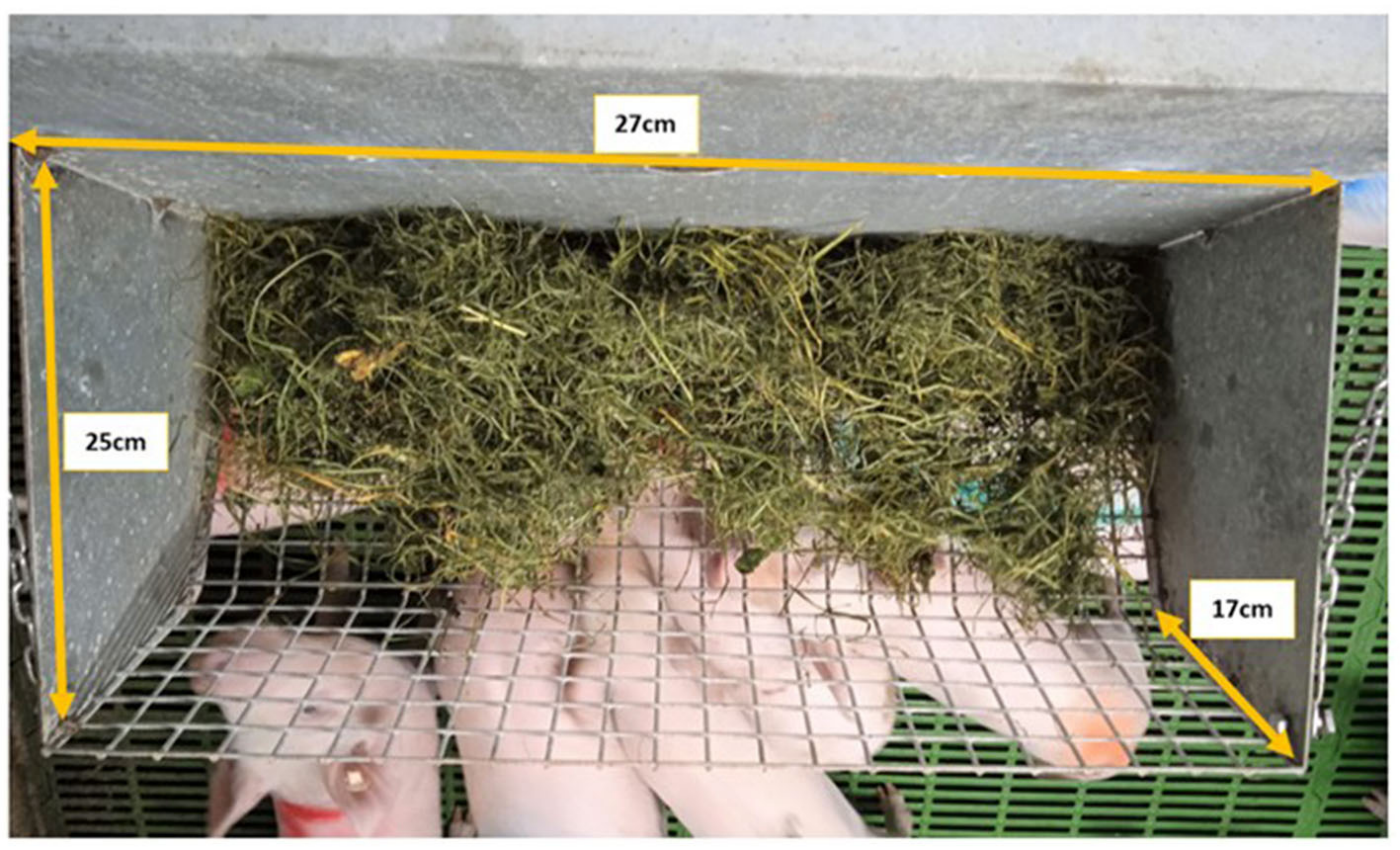
Rack dimensions (27 cm × 25 cm × 17 cm) for grass enrichment used from weaner to finisher stage for both SINGLE and DOUBLE.

Metal racks (0.59 × 0.26 × 0.25 m) were fitted on the front wall of each pen adjacent to the corridor (0.6 m above ground and 0.8 m from the feeder). Racks were 27 cm in length in both the weaner and finisher stages (Figure 2). The grass was added two times a day to ensure that pigs had *ad libitum* access. The weight of the grass was recorded whenever it was renewed, and the total sum for each pen during each stage (weaner and finisher) was calculated.

### 2.4. Physical measurements

#### 2.4.1. Lesions to the tail, ear, and flank

Every 2 weeks, tail, ear, and flank lesions were scored on each pig individually by a single observer. The tail lesions were scored using the Farewelldock Tail Scoring Protocol (25). Tails were scored for lesion severity (0 = no lesion, 1 = bite marks, 2 = open wound, 3 = swollen tail and bite wounds), the freshness of any blood present (0 = no damage, 1 = black scab or dry blood, 2 = older red blood, 3 = fresh blood), and tail length (0 = no cannibalism, 1 = partly shortened but tail >1 cm in length, 2 = tail <1 cm). Ear necrosis lesions were scored using the scoring system of Chou et al. (26): 0 = no lesions, 1 = black scab, 2 = evidence of recent bleeding, 3 = bleeding and reddish presence of cuts, and 4 = part of the ear missing. Ear scratches (long red lines, which sometimes broke the skin surface) were also scored using the following system: 0 = no lesion, 1= minor scratch presented on 1/3 of the ear, 2 = scratches presented on 2/3 of the ear, 3 = whole ear affected by scratches. Flank lesions were scored using the method detailed by Diana et al. (27): 0 = No evidence of flank biting, 1 = superficial circular lesion, blood and infection, 2 = larger circular lesion, blood and infection, 3 = extensive circular lesion, blood, and infection.

#### 2.4.2. Thermal image capture and analysis

Individual thermal images were recorded at the base of each pig's ears and tail at transfer from the weaner to the finisher stage (d47) using a FLIR T420 Infrared camera (thermal resolution: 320 × 240, measurement accuracy: ±2°C, thermal sensitivity: <0.04°C; FLIR Systems, Wilsonville, OR, USA). Before acquiring pictures, the reflected room temperature was measured as the mean temperature of a crunched aluminum foil (emissivity = 1). The room temperature was also recorded using the same room thermometer (LCD type min/max thermometer, Manotherm; sourced from Ark Animal Care, Newbridge, Ireland) at the time of each image acquisition. The skin emissivity of the pig was set at 0.98, as validated by Soerensen et al. (28). These parameters are important for the correct analysis of the thermal images, as they are used by the software to calculate the subject temperature. Each image was taken at a 1 m distance from each body part, at an angle of about 75° (28). Thermal images were taken when the animals were restrained in an individual weighing scale, thus allowing for a consistent distance and angle from the body parts.

Thermal images were processed with Thermacam Researcher Pro 2.0. Emissivity, reflected temperature, and room temperature were modified for each image so that calculated temperatures were accurate. An area was drawn around the base of both ears and the tail (Supplementary Figure S1B), and from this area, the maximum temperature was extracted. Data were then entered into an Excel file for analysis. Due to equipment malfunction, unfortunately, data were only available from Replicate 1 (*n* = 144 pigs).

#### 2.4.3. Pig performance

Pigs were weighed individually at weaning (d0), upon moving to the finisher house (d47), and before slaughter (d120). These data were used to calculate individual average daily gains (ADG) for both the weaner and finisher stages. From this, the coefficient of variation in pen weight was determined at each stage.

Feed delivery to each pen was recorded on a daily basis through the farm's computerized feeding system (BigFarmNet Manager, Big Dutchman Ltd. v3.1.5.51039, Vechta Calveslage, Germany). The amount delivered on each day was divided by the number of pigs in the pen (to take account of any removals due to illness, tail biting, etc.), then the average daily feed intake per pig (ADFI) was calculated for the weaner and finisher stages separately.

The entire pen weight at the beginning and end of each stage was used to calculate the pen average ADG. The feed conversion ratio (FCR) was calculated by dividing the ADFI by the pen average ADG.

#### 2.4.4. Post-mortem inspection

Pigs were individually marked with slap marks the day prior to slaughter and traced to the abattoir to obtain the post-mortem measurements from individual carcasses. Tails were scored on the slaughter line after scalding by a single trained observer, using the scoring system of Harley et al. (29) (0 = no lesion, 1 = healed/mild lesions, 2 = evidence of chewing and puncture wounds, 3 = signs of swelling and infection, 4 = partial/total loss of tail). The individual carcass quality report, including cold weight, the percentage of lean meat, muscle %, and fat % data, was obtained from the slaughterhouse.

### 2.5. Behavior measurements

#### 2.5.1. Group behavior

Animal behavior was observed at pen level every 2 weeks (*n* = 8 occasions; day 7, day 21, day 35, day 49, day 63, day 77, day 91, and day 105 of the experiment). Each pen was observed using all occurrences of continuous behavior recording for 4 × 5 min observation sessions (starting at 09:00, 11:30, 13:30, and 15:00) using the ethogram described in Table 1 [as per Chou et al. (26)]. A single observer carried out all observations, and the order of observation of each pen was randomized during each session over the course of the experiment. The rate of performance of each of the behaviors listed in the ethogram was calculated (instance/pig/5 min).

**Table 1:** Ethogram for all occurrence behavior observations at pen level.

**Behavior**		**Description**
Aggression	Fighting	Mutual pushing parallel or perpendicular, ramming or pushing of the opponent with the head, with or without biting in rapid succession
	Head knock	Pig swipes head vigorously and makes contact with it against recipient pigs body
	Body bite	Oral manipulation including bites directed toward parts of another pig
Damaging	Tail bite at the feeder	Tail in the mouth of a pig who is feeding: ranges from tail being gently manipulated to tail being chewed/bitten
	Tail bite	Tail in the mouth of another pig: ranges from tail being gently manipulated to tail being chewed/bitten
	Ear bite	Ear in the mouth of another pig: ranges from ear being gently manipulated to being chewed/bitten
	Belly nosing	Rhythmic up-and-down movement of the snout of one pig rubbing the belly of another
Enrichment	Interaction with toy/wood	Any form of oral/nasal manipulation of the rubber toy (weaner stage) or wood (finisher stage)
	Interaction with rack	Any form of oral/nasal manipulation of the rack that contained grass
Play	Play	Play behavior, scampering, jumping/running around

#### 2.5.2. Behavior at the feeder

All pens were video recorded using 2.0MP fixed wide angle bullet cameras with 40 m infrared night vision, HIKVision, China for a 4 × 24 h period, two times during the weaner stage (day 13 and 41), and two times during the finisher stage (day 62 and 111). Cameras recorded all pens within each replicate simultaneously, focusing on the feeder area. Recordings were downloaded onto a 1TB Hard drive (PC PRO Computers Ltd., Ireland). An hour of video footage was extracted for each feeder from 11:00 to 12:00 on each recording day, as preliminary observations of video over 24 h indicated that this is when pigs were most active at the feeder. Videos were transformed from .mp4 to .avi format using VSDC Free Video Editor (Multilab LLC). Observations were performed using The Observer XT (Ver. 14, Noldus, Wageningen, The Netherlands).

Two types of behavior were observed: feeding and competitive behaviors associated with feeding. A pig was considered to be feeding when its head entered the feeder space (snout positioned inside the rim of the feeder). In the DOUBLE treatment, whether the pig's head entered the IN or WALL feed space was also recorded. The number of feeding bouts, feeding bout duration, and the total occupancy of each feed space during the hour of observation were calculated, as well as the number of bouts overall and the total occupancy of the feeder (i.e., across both feed spaces in DOUBLE). Competitive behaviors were recorded when experienced by a pig that was feeding, and are described in Table 2.

**Table 2:** Ethogram for damaging behaviors experienced by pigs while feeding.

**Behavior**	**Description**
Tail bite	Tail in the mouth of a pig who is feeding: ranges from tail being gently manipulated to tail being chewed/bitten
Head knock	Pig swipes head vigorously and makes contact with recipient pigs body
Body bite	Oral manipulation including bites directed toward part of another pig
Belly nosing	Rhythmic up-and-down movement of the snout of one pig rubbing the belly of another
Displacement	The pig that is feeding removes its head from the feeder in response to being pushed by another pig
Mounting	Placing hooves on the back of another pig with or without pelvic movement
Pushing	Pushing the pig who is eating, without apparent interest in establishing social contact, to reach the feeder

All behaviors were recorded as frequencies.

### 2.6. Statistical analyses

Data were analyzed using SAS v 9.4. Unless specified, the pen was considered the experimental unit. Results were deemed statistically significant when α level was ≤0.05, and a tendency toward significance was considered when α level was >0.05 and ≤ 0.1. For all normally distributed continuous data, a linear general model was used to analyse the data (Proc Mixed). For these analyses residuals were examined to verify the normality and homogeneity of variances. Degrees of freedom were estimated using Kenwood–Rogers adjustment. Data are presented as LS means and standard errors, and the Tukey–Kramer adjustment is used for multiple comparisons. PROC UNIVARIATE was used initially for evaluating data distribution.

In all models, fixed effects included treatment (DOUBLE vs. SINGLE), recording day (where repeated measures were used), the interaction between these two measurements, and replicate (1 or 2).

#### 2.6.1. Physical measurements

Group ADG, ADFI, FCR, coefficient of variation of weight in the pen, enrichment weights, and thermography data were analyzed as mentioned earlier. For analysis of individual weights, ADG, and coefficient of variation of weight in the pen, data collected at weaning were also included in the model as covariates. Sex was considered a fixed effect for individual weights and ADG, and the pen was considered a random effect. Thermography images taken from the left ear, right ear, and tail base were analyzed separately using individual models for each.

All body lesion scores, including slaughterhouse tail scores, were analyzed using a generalized linear mixed model (Proc Glimmix) as the data were ordinal in nature. The sow was included as a random effect, and a multinomial distribution was specified. The model could not converge when data from all weeks were included as repeated measures, so each week was analyzed separately and *P*-values were adjusted *post-hoc* using a Bonferroni adjustment.

#### 2.6.2. Behavior measurements

Group-level behaviors were analyzed using a general mixed model (PROC MIXED). Behaviors were grouped into four categories: aggressive, damaging, interaction with the enrichment, and play (categorized in the Ethogram; Table 1). Behavior data were summarized per 5-min recordings and divided by the number of pigs present in the pen. Fixed effects included treatment, recording week, and the interaction between them. The recording week was also included as a repeated effect.

Feeding behavior was analyzed at two levels: individual feed space (IN and WALL) and total feeder level. For the former, feed space within treatment was also included as a fixed effect. For both levels, the number of pigs in the pen was included as a covariate. Feed bout duration was transformed using a log transformation so that the residuals approached normality.

Several of the aggressive behaviors performed around the feeder were sporadic when considered individually and did not approach a normal distribution. Thus, the data were summarized in three ways to provide three estimates of aggression. First, all aggressive behaviors were summed on the basis of feeder, and recording occasions, to give a total count. Second, all biting behaviors (belly, body, and tail bites) were summed, and finally, all non-biting behaviors were summed (displace, head knock, mount, and pushing). Displacements were also analyzed individually. All behaviors and non-biting behaviors were analyzed using a general linear model (Proc Mixed) and biting and displacements using a similar generalized linear model (Proc Glimmix), specifying a Poisson distribution. The rate of all aggressive behavior (no instances divided by the duration of feeding per feeder) was also analyzed using a linear mixed model. Data were log-transformed so that the distribution of the residuals approached normality.

## 3. Results

### 3.1. Tail-biting outbreaks

**Figure 3:**
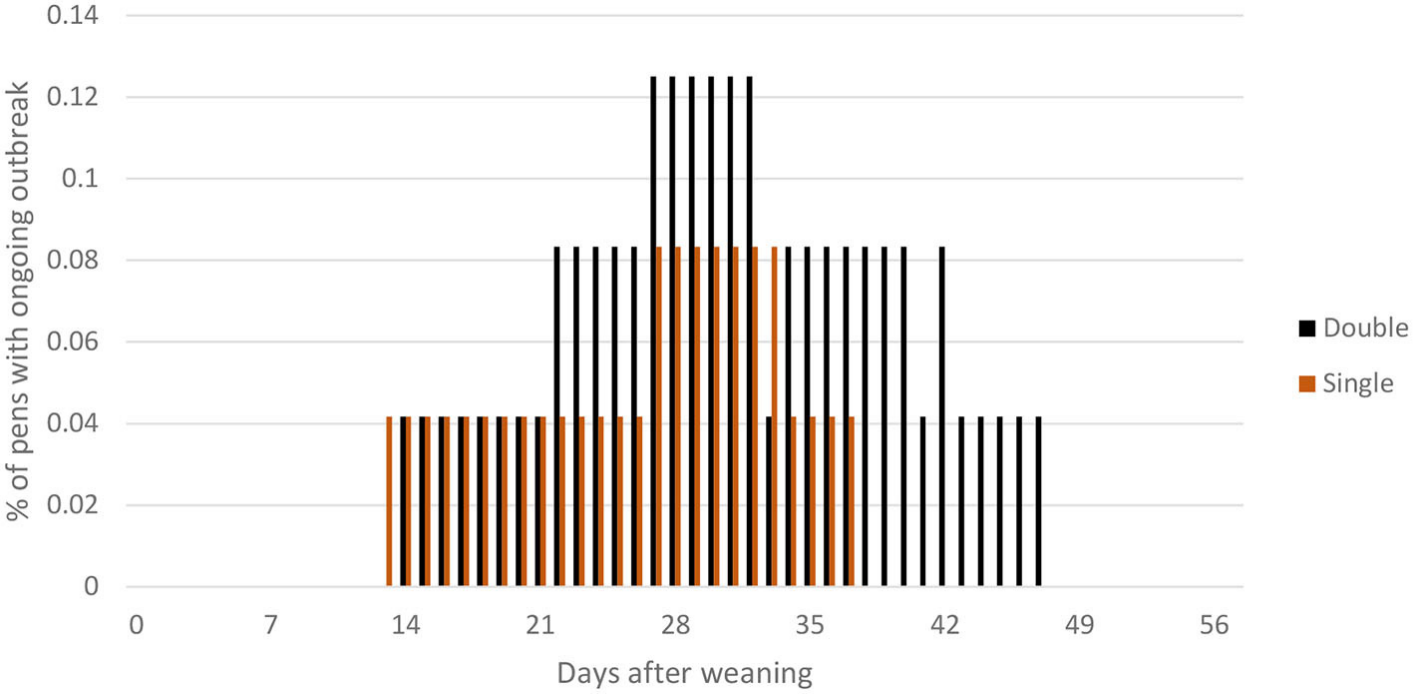
Percentage of pens with ongoing tail-biting outbreaks (defined as being between the criteria for the start of an outbreak and successful resolution, criteria for each of these are explained in the text) plotted against days post-weaning within DOUBLE and SINGLE.

During the course of the experiment, seven tail-biting outbreaks were recorded, two in SINGLE (d14, d29) and five in DOUBLE (day 14, day 22, day 28, day 33, and day 42; Figure 3). One pen (in DOUBLE) had a tail-biting outbreak relapse (day 14 and 33). Table 3 lists the number of pigs removed or treated in each treatment for infected tails (treated using an injection of antibiotics and analgesics) due to severe tail biting.

**Table 3:** Details of tail-biting outbreaks and severe tail damage (i.e., some level of tail amputation) during the experiment in each treatment, including the total number of pigs removed as tail-biting victims or biters, and treated by injection for tail-biting injury.

	**SINGLE**	**DOUBLE**
Number of outbreaks	2	5
Victims^a^	7	38
Biters	6	3
**Temporary removal**		
Victims	1	4
Biters	1	2
**Permanent removal**		
Victims	2	-
Biters	3	1

If a pig was removed repeatedly, the number was counted repeatedly in order to reflect the repeated tail biting events.

^a^Victims of tail biting who had lost part or the total of the tail during tail-biting outbreaks.

Six pigs were permanently removed from the experiment. Of these, two pigs were removed because they were experiencing severe tail lesions and four were due to being obsessive biters. In total, five individual pigs had tail wounds that necessitated temporary removal from their home pens for treatment (four in DOUBLE and one in SINGLE) and seven individual pigs were removed as tail biters. An additional nine pigs were treated with antibiotic injections in the home pen.

At the end of the experiment, a total of 90 pigs had some level of tail amputation, with 45 of these occurring due to tail-biting outbreaks. Of those, 38 were victims of tail-biting outbreaks in DOUBLE-space feeders' pens, and seven in SINGLE-space feeders' pens.

### 3.2. Physical measurements

#### 3.2.1. Enrichment consumption

The treatments had no effect on the consumption of enrichment materials (*P* > 0.05). In both stages, the total quantity of grass added to the racks was similar in both treatments (113.3 ± 11.6 kg, DOUBLE; 107.8 ± 11.6 kg, SINGLE; mean ± SE). Similarly, the consumption of rubber floor toys in the weaner stage (DOUBLE 0.17 ± 0.02 vs. SINGLE 0.13 ± 0.02 kg/day, mean ± SE) and the consumption of the plank of wood in the finisher stage (DOUBLE 0.44 ± 0.09 vs. SINGLE 0.26 ± 0.10 kg, mean ± SE) did not differ.

#### 3.2.2. Lesions to the tail, ears, and flank

Tail lesion results obtained on each test are described in Table 4.

**Table 4:** Percentage of pigs in each treatment that had a score >0 (perfect) for tail length, the freshness of blood, and damage of the skin of the tail, from the weaner stage to slaughter.

	**SINGLE**			**DOUBLE**		
	**Tail length**	**Tail damage**	**Freshness of blood**	**Tail length**	**Tail damage**	**Freshness of blood**
Week 1	1%	2%	4%	–	**–**	1%
Week 3	3%	3%	5%	5%	14%	14%
Week 5	4%	6%	4%	25%	32%	26%
Week 7	4%	1%	2%	29%	17%	15%
Week 9	6%	1%	1%	39%	12%	11%
Week 11	7%	13%	9%	39%	30%	24%
Week 13	12%	10%	8%	42%	27%	10%
Week 15	25%	8%	7%	43%	8%	4%
Week 17	35%	16%	7%	44%	13%	3%

Week refers to the week of the experiment, with the experiment starting at weaning (4 weeks of age).

If a pig presented a shorter length, skin damage, and the presence of blood repeatedly, the number was counted repeatedly in order to represent the change of events in the pen.

In general, values for all three tail lesion scoring systems rarely exceeded 1. Among these, 15 pigs' tails scored the highest in the length category (score 2), meaning their tails were shortened to < 1 cm after being a victim to biting (*n* = 2 SINGLE; *n* = 13 DOUBLE), 75 pigs' tails presented skin damage as an open wound and swollen and bitten tail (*n* = 30 SINGLE; *n* = 45 DOUBLE), and 78 presented fresh or older red blood (*n* = 35 SINGLE; *n* = 43 DOUBLE). At the first inspection, only one pig in the SINGLE treatment had a score above 0 for tail length, and as such this week was not included in the analysis. For the remainder of the experimental weeks, there was only an effect of treatment in week 6; more tails had some level of amputation in DOUBLE than in SINGLE.

With regard to skin damage, data for the first and last inspection did not converge when the pen was included as a random effect. When the calculated *P*-values for the remaining weeks were adjusted, there was no effect of treatment on any week. Finally, with regard to the freshness of blood, data from the fifth and final inspection did not converge. When the remaining *P*-values were adjusted, there was no effect of treatment on any week.

Necrotic lesions on the ears were only observed on eight occasions (DOUBLE = 7, SINGLE = 1), and as such were not statistically analyzed. There was no effect of treatment on ear scratch or flank lesion score on any week. Flank lesions were observed in only one pen in DOUBLE and three pens in SINGLE, and as such could not be analyzed statistically.

#### 3.2.3. Thermal image capture

The temperature at the base of the left and right ears were similar in both treatments (~37.1 ± 0.1°C for the left, and 37.3 ± 0.1°C for the right). However, pigs in DOUBLE had a lower temperature at the base of their tails than those in SINGLE (DOUBLE = 36.7 ± 0.12°C vs. SINGLE = 37.2 ± 0.12 °C; *P* = 0.01).

#### 3.2.4. Tail lesions and the presence of bruises at slaughter

When analyzing the results obtained at slaughter, no differences were detected for both tails' lesions score and the presence of bruises (*P* > 0.05).

#### 3.2.5. Animal performance

Table 5 summarizes the effect of treatment on animal performance. There was no effect on group live weight, ADG, or ADFI. However, the overall feed conversion ratio (FCR) was lower in DOUBLE than in SINGLE (*P* < 0.05), with DOUBLE tending to be lower in the weaner stage (*P* = 0.08).

**Table 5:** Differences (LS means ± SE) between treatments on measures of animal performance from day 28 to 120, for a total of 12 pens per treatment (12 pigs/pen), with an original weight of 8.6 ± 1.49 kg each pen.

	**Weaner**		**Finisher**		***P*-value**
	**SINGLE**	**DOUBLE**	**SINGLE**	**DOUBLE**	
Pig LBW, kg	32.3 ± 1.3	33.6 ± 1.2	104.8 ± 1.3	107.1 ± 1.2	0.272
Pig ADG, g/day	530 ± 20	550 ± 10	990 ± 10	1010 ± 10	0.229
Pig ADFI, g/day	810 ± 0.04	790 ± 30	2250 ± 40	2270 ± 30	0.915
Pig FCR, g/g	1.54 ± 0.03	1.43 ± 0.03	2.27 ± 0.03	2.27 ± 0.03	0.015

Single refers to pens with a single-spaced feeder (i.e., one feed space per 12 pigs). Double refers to pens with a feeder with two spaces (i.e., one feed space per six pigs).

#### 3.2.6. Carcass measures at slaughter

The use of double-space feeders had no effect on the carcass cold weight (*P* > 0.05). However, it had an effect on lean meat yield and kill-out yield, with pigs in SINGLE having higher values for both (*P* < 0.05; Table 6).

**Table 6:** Differences (LS means ± SE) between treatments in carcass quality at slaughter.

	**SINGLE**	**DOUBLE**	***P*-value**
Cold carcass weight, kg	79.42 ± 0.892	80.42 ± 0.864	0.421
Fat, mm	12.42 ± 0.224	12.91 ± 0.216	0.115
Muscle, mm	50.54 ± 0.623	49.27 ± 0.606	0.144
Lean meat, %	59.01 ± 0.165	58.52 ± 0.160	0.034
Kill out, %	0.75 ± 0.003	0.74 ± 0.003	0.040

### 3.3. Behavior measurements

#### 3.3.1. Group behavior

**Figure 4:**
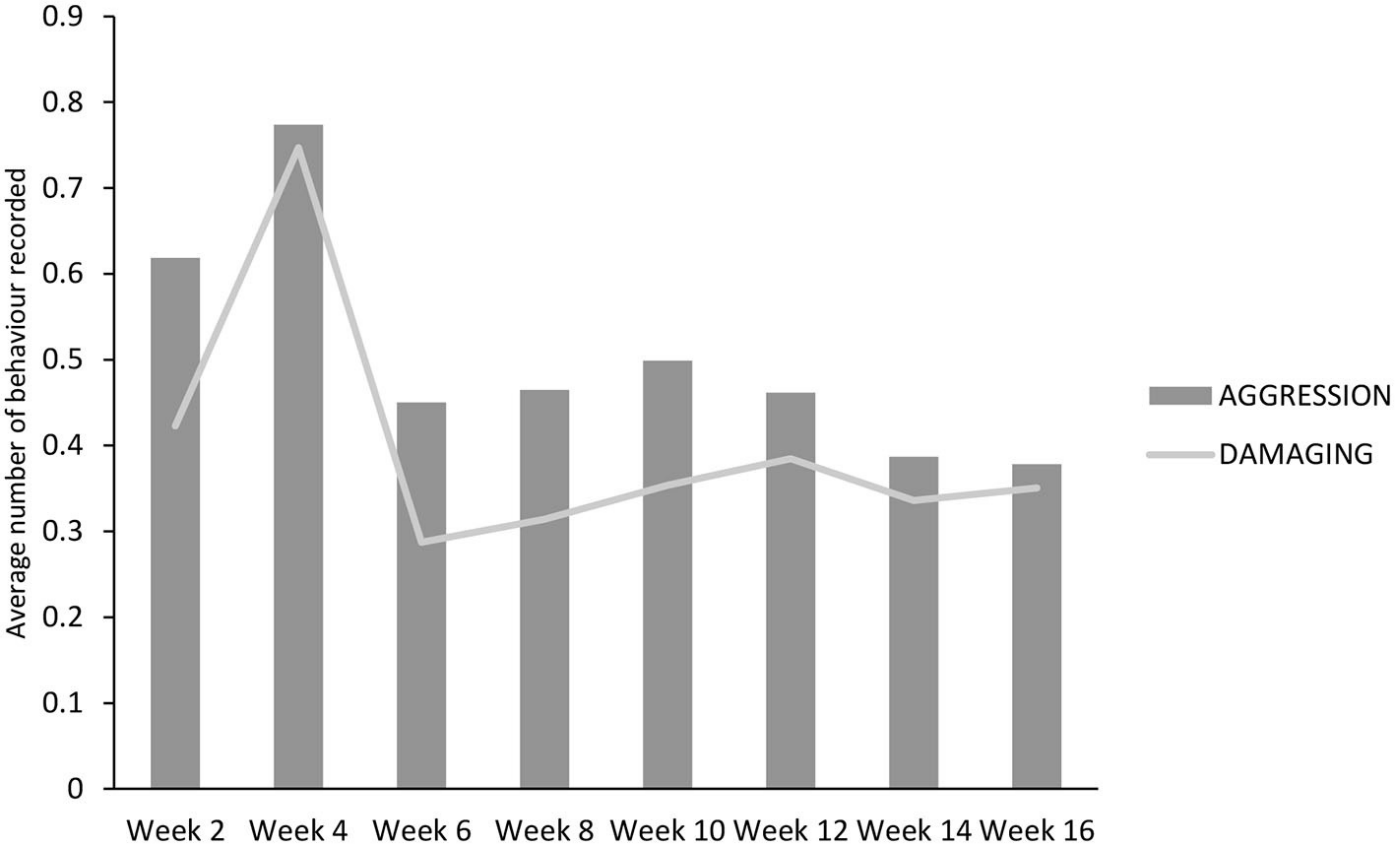
Aggressive and damaging behavior performed (instances/pig/5 min) during the course of the trial by both DOUBLE and SINGLE.

There was no effect of treatment on any of the behaviors recorded at the group level (Table 7), although there was an effect of inspection day for all (*P* < 0.01). Aggressive behaviors were highest and did not differ between weeks 2 and 4. However, they thereafter decreased, with week 2 being higher than weeks 14 and 16 (*P* < 0.05 for both) and week 4 being higher than all other weeks (*P* < 0.01 for week 10, *P* < 0.001 for all others; Figure 4). The same pattern was observed for damaging behaviors, although in this case, week 4 was higher than all others (*P* < 0.001; Figure 3). The maximum interaction with the enrichment materials was recorded in weeks 2, 4, and 6, and thereafter decreased until week 16. Similarly, as time progressed play behavior decreased.

**Table 7:** Differences (LS means ± SE) between treatments of the frequency of behaviors per pig recorded during direct observation of behavior at pen level over a 5-min period.

**Behaviours^a^**	**SINGLE**	**DOUBLE**	***P-*value**
Aggressive	0.51 ± 0.029	0.49 ± 0.028	0.678
Damaging	0.39 ± 0.021	0.41 ± 0.023	0.711
Enrichment	0.55 ± 0.032	0.49 ± 0.031	0.185
Play	0.08 ± 0.012	0.07 ± 0.011	0.657

a Number of instances of each behavior performed per pig per 5-min period.

### 3.4. Behavior at the feeder

#### 3.4.1. Feeding behavior

**Figure 5:**
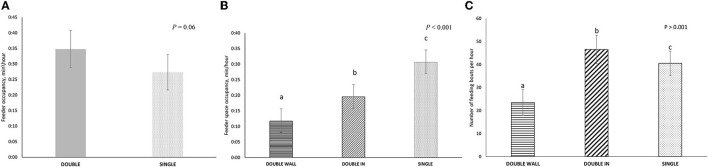
Feeding-related behavior in two treatments: (1) SINGLE, where pigs of twelve pigs had access to a single space feeder, and (2) DOUBLE where pens of 12 pigs had access to a double spaced feeder (i.e., one space per six pigs). Feed spaces in DOUBLE were classified as WALL (next to the front wall of the pen) or IN (adjacent to WALL, but on the inner side of the pen. Behavior was recorded continuously by video in each pen on four occasions for 1 h during the production cycle (i.e., 48 h of observation per treatment in total). **(A)** Total occupancy at the feeder in DOUBLE and SINGLE (min/h). **(B)** Total occupancy per feeder spaces (min/h) in DOUBLE WALL, DOUBLE IN, and SINGLE. **(C)** The total number of feeding bouts per hour in DOUBLE WALL, DOUBLE IN, and SINGLE. The different letters represent significance results.

The DOUBLE feeder tended to be occupied more than SINGLE (*P* = 0.06; Figure 5A). However, the average time that the feed spaces in DOUBLE (15:38 ± 03:50 min/h) were occupied over the entire hour of observation was about half of the time that the individual space in SINGLE was occupied (30:38 ± 03:42 min/h, *P* < 0.001). When the individual spaces were considered, both spaces in DOUBLE had less occupancy than the space in SINGLE (*P* < 0.001 for all comparisons, Figure 5B).

Although feed bout duration in DOUBLE was shorter than that in SINGLE (00:25 vs. 00:40 min/h; *P* < 0.001) there was no difference in duration between IN and WALL within the DOUBLE treatment. Overall, there were more feeding bouts in DOUBLE than in SINGLE (83.7 ± 10.4 vs. 42.9 ± 9.8 min/h; *P* < 0.001), with the number of feeding bouts influenced by the position of the feeder space; more bouts were recorded for IN rather than WALL (*P* < 0.001; Figure 5C). However, at the level of feed space, there was no difference in feed bout number between treatments.

#### 3.4.2. Competitive behavior

**Figure 6:**
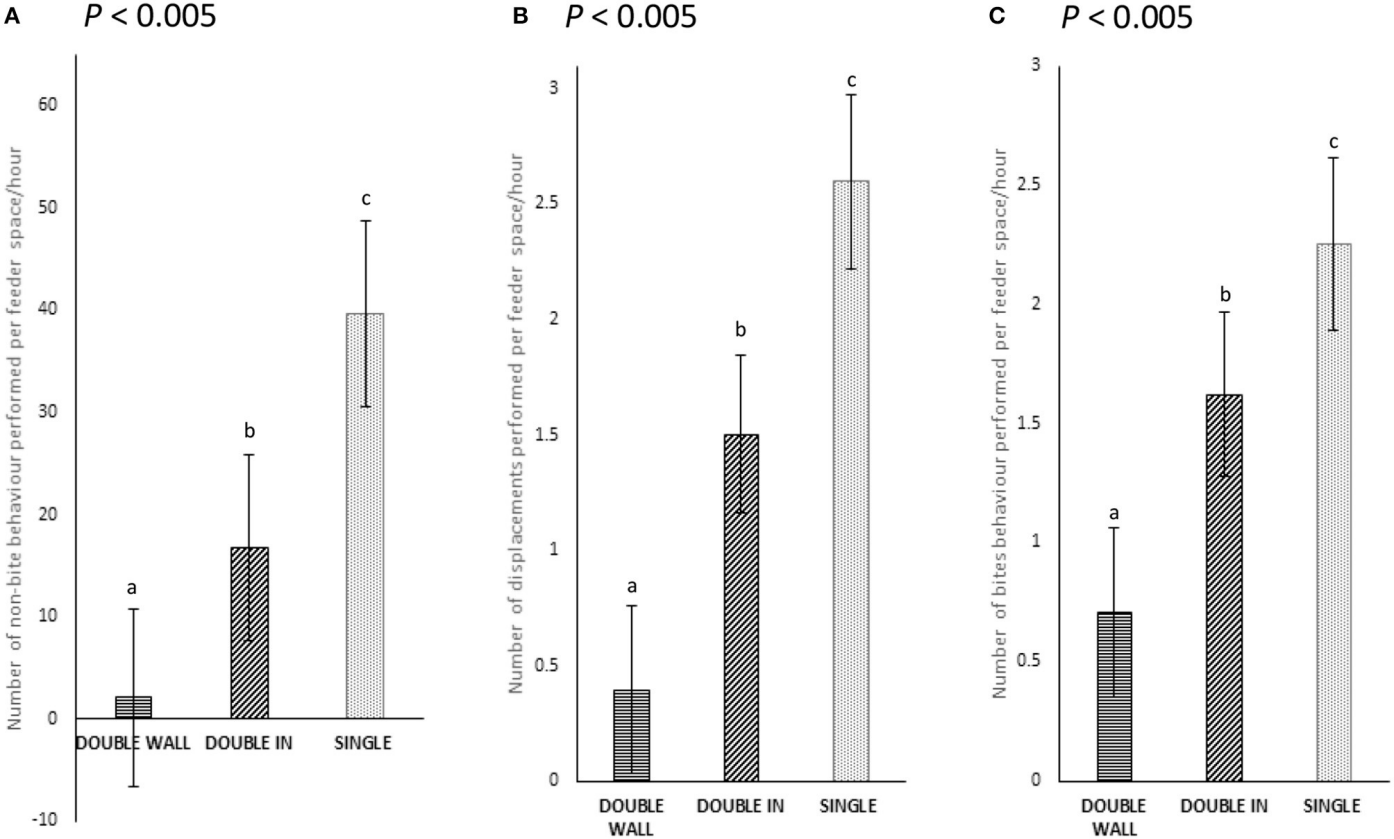
**(A–C)** The number of non-biting, biting, and displacements behaviors performed at feeder space (instances/min) in both SINGLE and DOUBLE (*P* < 0.005). The different letters represent significance results.

Pigs that were fed received fewer aggressive behaviors in DOUBLE (15.0 ± 9.8) than in SINGLE (52.8 ± 10.3; *P* < 0.001) pens. When considering feed spaces, there were fewer aggressive and displacement behaviors observed in both DOUBLE spaces (*P* < 0.01) than in SINGLE (*P* ≤ 0.001 for both comparisons). There was also a lower rate of performance of these behaviors in DOUBLE than in SINGLE (0.011 vs. 0.025 instances/min; *P* < 0.001). Similarly, a lower rate of performance for these behaviors was also detected in WALL than IN (0.009 vs. 0.014 instances/min; *P* < 0.005). In addition, when analyzing non-biting behavior, fewer behaviors were observed in DOUBLE than in SINGLE (*P* < 0.001), and fewer in DOUBLE WALL than in IN (*P* < 0.005, Figure 6A). Displacement behavior was also observed less in DOUBLE than in SINGLE (*P* < 0.001), and in DOUBLE, less often in WALL than in IN (*P* < 0.005, Figure 6B). Bite behaviors were also performed less in DOUBLE than in SINGLE (*P* < 0.0001) and again, fewer in WALL than in IN (*P* < 0.0001; Figure 6C).

## 4. Discussion

Competition for feed is considered one of the risk factors for the performance of aggressive and damaging behavior, and, consequently, could increase the risk of tail biting in pigs. Results of the present study provide some evidence that increasing feed space allowance can effectively help reduce aggressive behavior when pigs are feeding, although this management strategy did not influence the typical behavioral pattern at the pen level, nor did it have a protective effect when it came to tail damage.

Although the numbers were too low for statistical comparison the number of tail-biting outbreaks observed during the experiment was greater in DOUBLE than in SINGLE. A consequence of this is that the number of animals involved in tail biting, and tail damage recorded *via* routine lesion scoring was higher in DOUBLE than in SINGLE. These results showed that although double feeders allow for more feeder space, it still does not allow group feeding. This is in contrast to studies that suggest that increasing feed space may help reduce the possibility of this behavioral problem and avoid the possibility of a tail-biting outbreak occurring (30, 31). Nevertheless, the severity of damage was low in both treatments, with only 15 pigs in total experiencing more than the loss to the tip of the tail.

Although tail-biting outbreaks occurred only during the weaner stage, tail-directed behaviors were yet observed, and fresh tail lesions were identified until the end of the study. Overall, the severity for most of the tail bites identified was non-severe and, therefore, did not result in damage at the pen level that met the threshold to be considered a tail-biting outbreak. Nevertheless, DOUBLE pigs experienced more severe consequences from biting behavior in terms of tail length (i.e., a score of >1) than SINGLE. It is highly probable that the treatment effect during week 6 can be attributed to the time these data were collected, 1 week after a tail-biting outbreak in the DOUBLE treatment. Moreover, once part of the tail is bitten off, there is no recovery to a lower score, so the effect over time is cumulative. By the end of the study, the percentage of tails that were not intact in both experimental groups was higher in DOUBLE than in SINGLE. However, in DOUBLE, the rate of increase in the percentage of pigs that had partial or complete missing tails increased very slowly from Test 5 to the end of the study. On the contrary, it increased in SINGLE. This could imply that the level of the missing tail in DOUBLE derived from the tail-biting outbreaks early during the pig production cycle; meanwhile, the severe tail damage in SINGLE continued to accumulate over the lifetime of the pig on the farm. It is, thus, surprising that the thermal image capture showed that the temperature at the base of the tail was higher in SINGLE compared to DOUBLE, particularly as this was taken at the end of the weaner stage, just after the main period when tail-biting outbreaks occurred. The thermal image capture was used to detect whether there was an alteration of body temperature on the tail or ear derived from biting or other damaging behavior. However, there are many factors that can impact body temperature, including simply the rate of movement; it is possible that if pigs in DOUBLE were moving their tails more to evade being bitten, or if they were sensitive, this could have slightly raised the temperature. Elevated body temperature can also result from an inflammatory reaction, especially in a controlled environment such as a commercial pig farm. Indeed Teixeira et al. (32) found that as damage to the tail increased, so did the skin temperature at the base of the tail. However, in that study, the greatest increases were in tails that had clear, severe, damage. In contrast, in the current study, the damage was minor, and the difference in temperature was only 0.5°C, so not likely to have been biologically significant.

Overall aggressive and damaging behavior, detected at the pen level, was similar in both DOUBLE and SINGLE feeders, in contrast with what was suggested by other authors, who have reported that increasing feeders' space could help reduce both types of behavior in pigs raised in an intensive indoor system (23). However, during this trial, aggressive and damaging activity decreased over time from week four, which does correlate with what has been reported by other authors, who reported a similar pattern when investigating behaviors on pigs from grower to finishing (33, 34). Thus, the results appear to be externally valid, as overall pigs expressed a pattern of behavior over time that is consistent with previous studies.

The effectiveness of the DOUBLE-space feeders in reducing the amount of aggressive and damaging behaviors that pigs experience while feeding was confirmed by the results from the video observations. Pens with SINGLE-space feeders had a greater level of feeders' space occupancy, and pigs experienced more aggressive behavior during both growing and finishing periods than those in DOUBLE. This suggests that in these pens there was a greater level of competition for the feeder (35). This result is in line with what was reported by Andersen et al. (36), who suggested that competition for feed increases when the feeder space decrease and group size increase (37). The decrease in the accessibility of the feed in SINGLE likely influenced not only the feeding behavior of the animals in SINGLE, who were sometimes displaced but also the behavior of the animals that were not feeding, when they were attempting to gain access to the feeder.

In the present experiment, a SINGLE- or DOUBLE-space feeder was shown to affect pigs' efficiency. Although average live body weight, daily gain, and feed intake were not significantly different in the two treatments, the numerically higher live weight resulted in a statistically lower feed conversion ratio. However, the feed intake remained numerically very similar. These results suggested that pigs reared in less competitive circumstances have a better FCR, as demonstrated in previous studies (38, 39). Pigs from SINGLE had a higher kill-out percentage and higher lean meat percentage than those in DOUBLE, which is in contrast with previous studies that have demonstrated that kill-out percentage increases as slaughter weight increases (40). In addition, it is in contrast to studies that suggest that improving animal welfare allows pigs to reach their maximum potential for lean deposition (38, 39).

To the best of our knowledge, only a small number of studies have been published regarding the preferred position of the feeder space for pigs from weaner to slaughter age (41). To optimize space in the pen and provide sufficient space for moving and resting, feeders are usually placed on a sidewall or in the corner of the pen (41). However, our results showed that even when located adjacent to each other there can be significant differences in feed space use. Pigs in DOUBLE preferred to feed at the IN space feeder than those in the WALL. Bus et al. (37) suggested that when multiple space feeders are present, pigs could prefer a specific feeding space and are willing to wait for this rather than simultaneously feeding using the available space feeder. Although not objectively scored, it was noticed while watching the videos that pigs tended to change position while feeding frequently, and that their bodies tend to position themselves diagonally toward the inside or outside of the pen. Thus, the feeder that was located further inside the pen may have been preferred because it allowed the animals to assume favored positions more easily. In addition, while assuming these positions, the body of the feeding pigs can block access to the second feeder space, which leads to less occupation of the blocked feeder and, consequently, could lead to more aggressive and damaging behavior, which reduces the benefit derived of having a second feeder space.

## 5. Conclusion

This experiment demonstrated that doubling the feeding space from one space per 12 pigs to one space per six pigs allowed greater feeder occupancy and reduced aggressive and damaging behavior, indicating reduced competition for feed in both the weaner and finisher stages. However, this management strategy alone did not reduce tail-biting behavior relative to pens with the standard feeder.

Nevertheless, this study demonstrated that increasing feeder space could lead to better feed efficiency but does not improve the carcass quality at slaughter. Further studies need to be conducted regarding feeder positions and space allowance for pigs.

## Data availability statement

The raw data supporting the conclusions of this article will be made available by the authors, without undue reservation.

## Ethics statement

The animal study was reviewed and approved by Teagasc Animal Ethics Committee (TAEC2020-259).

## Author contributions

RD'A and KO'D contributed to the conception and design of the study, planned and organized the article, and performed the statistical analysis. RD'A drafted the manuscript. AH and KO'D revised the manuscript. All authors contributed to the article and approved the submitted version.
