# Supplementary figures and images for: Comparison of single- and double-spaced feeders with regard to damaging behavior in pigs
Source: Front Vet Sci. 2023 Feb 22;10:1073401. doi: 10.3389/fvets.2023.1073401 (PMC9992529; doi:10.3389/fvets.2023.1073401)

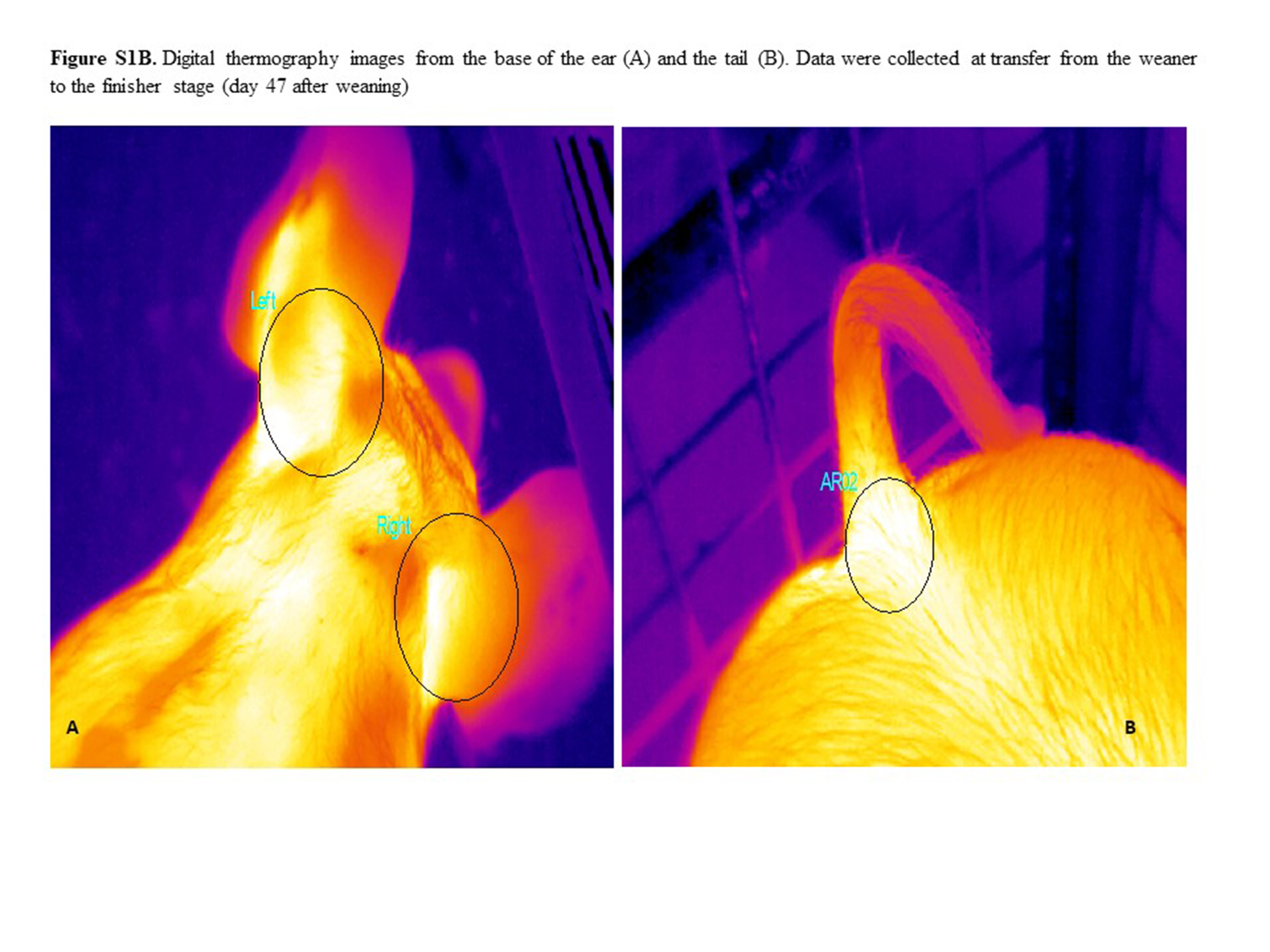

Supplement: Supplementary file 1 [file Image_1.JPEG]
